# The association between benzodiazepine co-prescription, opioid agonist treatment and mortality: a systematic review

**DOI:** 10.1186/s12888-024-06191-3

**Published:** 2024-10-28

**Authors:** Christine Hillestad Hestevik, Line Holtet Evensen, Hege Kornør, Ivar Skeie

**Affiliations:** 1 The Norwegian Institute of Public Health, Postboks 222 Skøyen, Oslo, 0213 Norway; 2 National Advisory Unit on Concurrent Substance Abuse and Mental Health Disorders, Department of Mental Health, Innlandet Hospital Trust, P.O. Box 104, Brumunddal, 2381 Norway

**Keywords:** Opioid agonist treatment (OAT), Opioid maintenance treatment (OMT), Medication assisted treatment (MAT), Benzodiazepine prescription, Mortality

## Abstract

**Background:**

Opioid agonist treatment (OAT) is the preferred treatment for opioid dependence due to benefits such as treatment retention, reduced opioid use and mortality. Benzodiazepine co-dependence is common in OAT patients and has been linked to increased mortality. Prescribing benzodiazepines during OAT has been tried to reduce the harms of extra-medical benzodiazepine use. This systematic review examines association between benzodiazepine co-prescription during OAT and mortality.

**Methods:**

We searched MEDLINE, Embase, Psych INFO, Cochrane Database of Systematic Reviews, Cochrane Central Register of Controlled Trials and Epistemonikos for reports published from database inception to June 2021. The searches were updated in February 2024. We included studies comparing mortality rates in OAT patients with and without benzodiazepine co-prescription. Two reviewers independently screened, extracted data, and assessed risk of bias from eligible studies with the Risk Of Bias In Non-randomized Studies of Interventions (ROBINS-I) tool. We combined the effect estimates in meta-analyses where possible. The certainty of the pooled effect estimates was assessed using the GRADE approach.

**Results:**

We included six observational studies (*N* = 84,452) conducted in Sweden, Scotland, Canada, England, and the USA. Moderate-certainty evidence linked benzodiazepine prescription to higher all-cause mortality on OAT (HR 1.83; 95% CI 1.59 to 2.11). Moderate-certainty evidence associated benzodiazepine prescription with higher non-drug-induced mortality during OAT and the whole observation period (HR 1.73; 95% CI 1.33 to 2.25) and HR 2.02; 95% CI 1.29 to 3.18). Low-certainty evidence suggested an association with higher drug-induced mortality on OAT (HR 2.36; 95% CI 1.38 to 4.0). Very low-certainty evidence linked benzodiazepine prescription to higher all-cause and drug-induced mortality throughout the observation period (HR 1.49; 95% CI 1.02 to 2.18 and HR 2.19; 95% CI 0.80 to 6.0).

**Conclusions:**

There is probably an association between prescribed benzodiazepine use and higher risk of all-cause mortality (on OAT) and mortality due to non-drug-induced causes (on OAT and on and off OAT). Benzodiazepine prescription may also be associated with higher all-cause mortality (on and off OAT) and drug-induced mortality (on OAT and on and off-OAT), but this is highly uncertain due to methodological issues and possible confounding.

**Supplementary Information:**

The online version contains supplementary material available at 10.1186/s12888-024-06191-3.

## Background

Opioid agonist treatment (OAT), also known as opioid maintenance treatment, is the most widespread and in many countries the first-choice treatment for opioid dependence [1–5]. The basic objective of OAT is to replace harmful extra-medical use of opioids with prescription of long-acting full or partial opioid agonists (mainly methadone or buprenorphine) to stabilize the patient without symptoms of abstinence or intoxication. Other important goals of OAT are to improve health and social functioning (such as housing, integration in drug-free networks, contact with family, education and employment). The medicinal treatment is usually combined with psychosocial support and rehabilitation services.

OAT patients have better retention in treatment, reduce their illicit opioid use and have fewer negative outcomes compared to patients in non-medicinal treatment and to people with opioid dependence who are not in treatment [6–9]. Further, it is well established that being on OAT is associated with a substantial reduction of all-cause and drug-induced mortality compared with not being on OAT [10–12]. Benzodiazepine co-dependence is common in OAT patients. In cases where tapering benzodiazepines or achieving abstinence has been unsuccessful or deemed unrealistic, prescribing benzodiazepines has been used as a harm-reducing strategy. Clinical experience and some studies suggest that such an approach may be appropriate for some patients [13, 14], but the evidence is weak. Due to the risk of life-threatening respiratory depression and death associated with the concomitant use of opioid agonists and benzodiazepines [15–17], guidelines recommend using such a strategy with caution [1, 4].

Providing high-certainty evidence for the association, and especially a causal link, between OAT/benzodiazepine co-prescription and mortality in OAT populations is a complex task. This challenge arises from a variety of factors such as polysubstance use, varying dosages, population heterogeneity, risks of misuse, ethical constraints in research, and data limitations. A recent study from Australia investigated mortality in people with opioid dependence who were prescribed benzodiazepines and found that all-cause mortality was reduced when on OAT compared to off OAT [18]. Although this was a study of a cohort of opioid dependent people and not an OAT-cohort, it may indicate an on-OAT mortality-reducing effect among opioid dependent people using benzodiazepines. In Scotland, measures to reduce the impact of benzodiazepine prescription on the number of overdoses may have had unintended and paradoxical effects [19]. Over the last 20 years, there has been an over 400% rise in overdose deaths [20], with concomitant use of opioids and benzodiazepines playing a major role. When benzodiazepine prescribing was restricted due its suspected impact on the rising overdose mortality, many dependent benzodiazepine users converted from prescribed to illicit use of benzodiazepines [19]. There was also a shift on the illegal benzodiazepine market from grade benzodiazepines diverted from medical prescriptions to illegally produced, often more potent and sometimes contaminated “street benzos” [19, 20]. This may have had a significant negative impact on the benzodiazepine contribution to the overdose epidemic in Scotland [19, 20]. The impact of new psychoactive substance (NPS) benzodiazepines on the illicit drug market is not limited to Scotland [21, 22]. OAT programme characteristics, such as specialist addiction medicine units vs. general practitioners, and how closely OAT patients are followed up, can also affect both benzodiazepine prescription practices and outcomes [13, 14, 23, 24].

Furthermore, it is challenging to determine the extent to which benzodiazepines contributed to the cause of death and whether they were used as prescribed or extra-medically. Findings from a recent Norwegian study suggest that illicit benzodiazepine use may play a bigger role than prescribed benzodiazepines in overdose deaths among OAT patients [25]. Finally, the mortality-reducing effect of OAT is mainly an on-treatment effect, and the ability of OAT programs to retain patients on treatment over long periods of time is very important. Therefore, it is insufficient to assess the effect of OAT programs on mortality simply on findings *on* OAT, it is crucial to examine mortality in an on/off OAT perspective to account for all participants, including those who drop out or relapse, providing a more accurate reflection of the programs’ long-term impact on mortality [26]. Regarding OAT patients who use benzodiazepines, it is just as important to study mortality in an on/off-OAT perspective as to compare them with OAT patients who do not use or are not prescribed benzodiazepines.

The Norwegian Institute of Public Health (NIPH), commissioned by the Norwegian Directorate of Health, carried out a systematic review in 2021 on benzodiazepine co-prescription on OAT, and its impact on mortality [27]. The review includes six observational studies, and meta-analyses results suggest an association between benzodiazepine prescription and mortality, both on OAT and during the entire period of observation. This review has only been published in Norwegian, and we believe that this topic would be of interest to the global society of researchers, clinicians, and policymakers within the OAT field. Therefore, we have updated the original review in English.

## Methods

We conducted a systematic review according to the guidelines described in Cochrane handbook [28] and to the Preferred Reporting Items for Systematic Reviews and Meta-Analyses (PRISMA) guidelines [29]. The aim of this systematic review was to investigate the association between prescription of benzodiazepines to persons on OAT and the risk of mortality. This review was an update of a previous review conducted by NIPH and therefore a review protocol was not prepared. The original protocol (in Norwegian) can be accessed here: Protocol.

### Search strategy

The original literature search was conducted in June 2021. A research librarian planned and conducted systematic searches in six electronic databases (MEDLINE, Embase, Psych INFO, Cochrane Database of Systematic Reviews, Cochrane Central Register of Controlled Trials and Epistemonikos). We updated this search in February 2024. In addition, we conducted a search in OpenAlex [30] which is a comprehensive repository of the World’s research and contains more than 200 million records. We accessed OpenAlex through the systematic review software EPPI-Reviewer [31]. We used the six studies that were included in the original report as seed studies to search for new relevant studies. The search period was January 2021 to February 2024. The search strategy is described in Appendix 1. We also consulted topic experts about relevant studies.

## Selection criteria

Eligible studies were randomized trials and non-randomized studies (e.g., cohort and quasi-experimental studies) comparing the prescribing of benzodiazepines (ATC codes N03AE01, N05BA01-19, N05BA21-24, N05BA56, N05CD01-15) with no benzodiazepine prescribing, including no treatment, placebo, other pharmacological, or non-pharmacological treatment in OAT patients. To be eligible, studies were also required to report on the following outcomes: All-cause mortality, drug induced mortality (regardless of type of drug), mortality due to non-drug-induced causes, as well as data source and duration of follow-up. There were no restrictions regarding publication year, country, or language.

The European Union Drugs Agency (EUDA) defines “drug-induced deaths” as: *‘deaths happening shortly after consumption of one or more illicit psychoactive drugs*,* and directly related to this consumption*,* although they often may happen in combinations with other substances such as alcohol or psychoactive medicines. Usually these deaths are also named ‘overdoses’*,* ‘poisonings’ or ‘drug-induced deaths ’* [32]. However, the studies included in this review used varying terms and definitions for this outcome. For the purpose of this review, we were obligated to follow the definitions used by the original studies, as outlined in Appendix 2, but refer to this outcome as ‘drug-induced deaths’ [32].

## Study selection

We used the machine learning function “priority screening” in EPPI-Reviewer [31] to make the screening titles and abstracts more efficient. The machine learns the characteristics of included and excluded studies and predicts whether a given reference is more likely to be relevant or irrelevant. Priority screening ‘pulls’ relevant records towards the beginning of the screening process and ‘pushes’ irrelevant ones towards the end. Titles and abstracts were independently screened by two authors to assess whether they met our inclusion criteria.

We then obtained references that we considered relevant in full text and two authors independently reviewed these and made a final assessment of which studies to include.

Discrepancies in decisions on the relevance of titles and abstracts and full texts were resolved by discussion among the authors.

## Risk of bias

To assess the risk of bias in the retrospective cohort studies and one case note review, we used the Risk Of Bias In Non-randomized Studies of Interventions (ROBINS-I) [33] tool. This tool has questions within seven domains: bias due to confounding, selection bias, bias in classification of interventions, bias due to deviations from intended interventions, bias due to missing data, bias in measurement of outcomes and reporting bias. The overall risk of bias for each study outcome was classified as either low, moderate, serious, or critical risk of bias. Confounders are factors that influence both the likelihood of participants receiving a specific intervention and the outcome itself. To assess bias due to confounding, we collaborated with a clinical expert and identified five important confounding factors: age, sex, comorbidity, psychiatric diagnosis or prior psychiatric treatment, and severity of substance use disorder.

To assess the risk of bias in the case-control study, we used the Newcastle-Ottawa Scale (NOS) for case control studies [34]. This tool has eight questions within three main domains: Selection, comparability, and exposure. We rated the overall quality of the studies as low, moderate, or high as suggested by the Conducting Systematic Reviews and Meta-Analyses of Observational Studies of Etiology (COSMOS-E) guidance [35].

## Data extraction

One author (CHH) extracted data from the included studies and another (LHE), checked the data against the publication.

We extracted the following data from the included studies:

- Bibliographical data (author, year, journal).
- Country.
- Information on participants and treatment groups (gender, age, benzodiazepine addiction status, OAT medication).
- Information about the treatment (type of benzodiazepine preparation, number of treatments/doses, duration of treatment and observation time).
- Death: All-cause mortality, drug-induced mortality (regardless of type of drug), mortality due to non-drug-induced-causes.

If a study had more than one publication, we extracted data from the publication that was most relevant for our outcomes.

### Analysis

We pooled the results from the included studies in meta-analyses, if appropriate (i.e., when studies were sufficiently homogeneous in terms of study design, participants, intervention, comparison, observation period and choice of analysis). We performed separate analyses for the outcomes all-cause mortality, drug-induced mortality, and mortality due to non-drug-induced causes. We performed analyses of periods on OAT and entire observation periods, respectively. Observation periods included both on-OAT and off-OAT episodes, however the definition of on and off treatment varied between the included studies.

Most of the included studies reported hazard ratios (HR) as the effect estimate. We log-transformed the HRs and performed meta-analyses using the inverse variance method and the random effects model. We also calculated 95% confidence intervals (CIs) for each effect estimate. The meta-analyses were illustrated using forest plots showing the HRs in an exponentiated format, that is, in the format reported by the primary studies. In the main analysis, we combined studies, although they did not consider the same confounding factors in the analyses. We intended to conduct sensitivity analyses excluding studies that only reported unadjusted effect estimates, but this was not done as only one unadjusted study [36] was included in a meta-analysis of two studies [36, 37]. Statistical heterogeneity was assessed with I^2^.

One of the included studies had not processed the data statistically, and only stated mortality rates per 100 person-years of treatment. For this study, we calculated an unadjusted rate ratio with a 95% CI based on the number of deaths per group and the number of person-years in treatment per group.

In most of the included studies, it was not possible to extract observation time (person-years) at the relevant group level. Thus, it was not possible to re-express results from the meta-analyses using absolute effect measures (i.e., mortality per 100 person-years).

All analyses were carried out in the software Stata version 17 [38].

## Grade

We used The Grading of Recommendations Assessment, Development and Evaluation (GRADE) approach [39] to assess our certainty in the effect estimates within the following five domains: risk of bias, inconsistency, indirectness, imprecision, and publication bias. The GRADE approach specifies four levels of certainty: high, moderate, low, or very low.

## Results

**Fig. 1:**
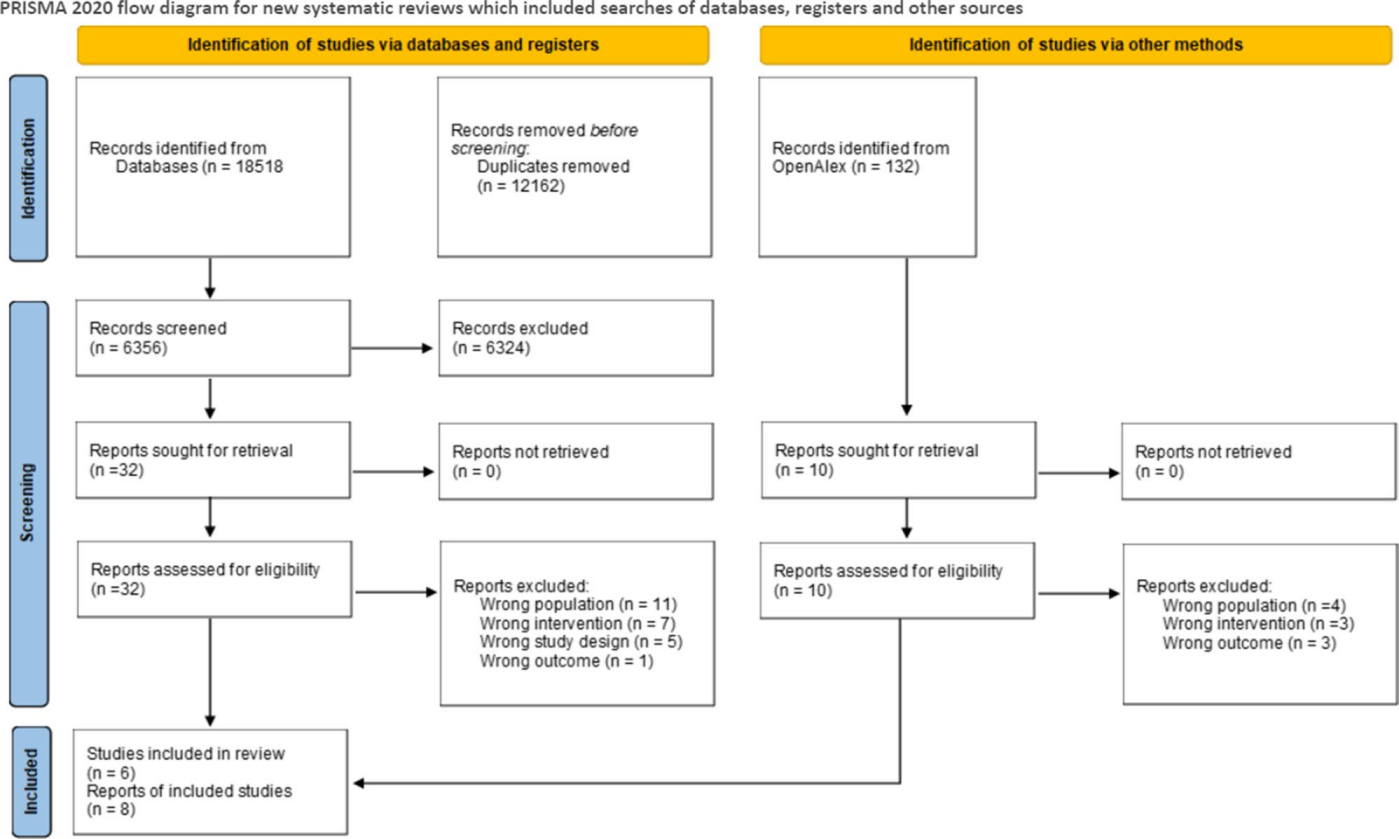
Prisma flow diagram for selection of studies

The first database search resulted in 5,154 references after duplicates were removed (Fig. 1). Of these we excluded 5,126 references that obviously did not meet our inclusion criteria. We reviewed 28 publications in full text of which we excluded 20. The updated search resulted in 746 references of which 14 studies were reviewed in full text. No new studies were included as none of these met our inclusion criteria. A list of excluded studies with reason is presented in Appendix 3. In total we included six studies reported in eight publications [14, 17, 36, 37, 40–43].

The included studies were published between 2009 and 2019. The studies were carried out in England [14, 42, 43], Scotland [36, 40], Sweden [37], Canada [41], and USA [17]. Five of the studies were retrospective cohort studies and one was a case-control study (Table 1). Five of the studies obtained data from health registries while one study was a review of medical records from a clinic. Only one of the studies examined prescription of benzodiazepines as treatment for benzodiazepine dependence [14].

**Table 1:** Description of the included studies

Study, year (country)	Study design	Participants and setting	Benzodiazepine prescriptions
Abrahamsson 2017 (38) (Sweden)	Retrospective register-based cohort study	*N* = 4,501 Gender: 74% men Age: median 34.4 years (IQR 28.7–42.1) OAT drugs: methadone or buprenorphine Follow-up time: mean 4.8 years Setting: OAT clinics approved by the NBHW	*N* = 1,458 Benzodiazepines: Diazepam, oxazepam, lorazepam, alprazolam, nitrazepam, flunitrazepam, triazolam, midazolam and clonazepam
Bakker 2017 (14) (UK)	Case-note review	*N* = 278 Gender: 69% men Age: no information Follow-up time: 1,289 patient years OAT drugs: methadone, buprenorphine, or slow-release morphine Setting: Central London general practice	*N* = 207 Benzodiazepines: not specified
McCowan* 2009 (37) (Scotland)	Retrospective register-based cohort study	*N* = 2,378 Gender: 65% men Age: 16–60 years (65% under the age of 30) Follow-up time: median 4.38 (IQR 1.92–8.12) years Setting: Residents in Tayside, who were registered with a general practitioner and were prescribed methadone between January 1993 and February 2004	*N* = 1,794 Benzodiazepines: not specified
Leece 2015 (42) (Canada)	Nested case-control study	*N* = 1,048 Gender: 62% men Age: median for intervention group 42 (36–48) years and control 39 (IQR 31–45) years Setting: methadone recipients in Ontario between 1994–2010 Follow-up time: not specified	*N* = 116 Benzodiazepines: not specified
Macleod* 2019 (44) (UK)	Retrospective register-based cohort study	*N* = 12,118 Gender: 67% men Age: 38.8 (SD 10.4) OAT drugs: Methadone and buprenorphine Follow-up time: mean 3.4 years. Setting: Persons on OAT who were registered in CPRD between 1998–2014	*N* = 5,114 Benzodiazepines: not specified
Park 2019 (17) (USA)	Retrospective register-based cohort study	*N* = 63,345 Gender: 62% men Age: mean 38 (SD 11) Follow up time: 1 day to 4 years Setting: People who were prescribed buprenorphine or buprenorphine/ naloxone between January 2012 and December 2015 as recorded in the Massachusetts PMP.	*N* = 15,223 Benzodiazepines: alprazolam, chlordiazepoxide, clonazepam, clorazepate, diazepam, estazolam, flurazepam, lorazepam, oxazepam, prazepam, quazepam, temazepam and triazolam.

IQR - interquartile range; OAT- opioid agonist treatment; SD - standard deviation; NBHW - Swedish National Board of Health and Welfare; CPRD - Clinical Practice Research Datalink; PMP - Prescription Monitoring Program. * In two studies, we identified more than one publication. For the Scottish study, two publications were identified: McCowan et al. [37] and Cousins et al. [41]. Data were extracted from the publication by McCowan et al. In the English study conducted by Macleod et al. [44], a journal publication was based on a report from the National Health Service (NHS), authored by Steer et al. [43]. Data were extracted from Macleod et al.‘s publication [44]

The studies included a total of 84,452 (278 to 63,345) participants in opioid agonist therapy (methadone in two studies, buprenorphine in one study and both drugs in three studies, one of which also included long-acting oral morphine). The participants’ age ranged from 15 to 96 years and the majority were men.

All studies compared mortality in OAT patients with or without benzodiazepine prescription. The length of the observation period varied between studies. In one of the included studies, Leece 2015 [41], we were unable to determine whether benzodiazepines were prescribed concurrently with OAT. See description of studies in Table 1.

### Risk of bias in the included studies

**Fig. 2:**
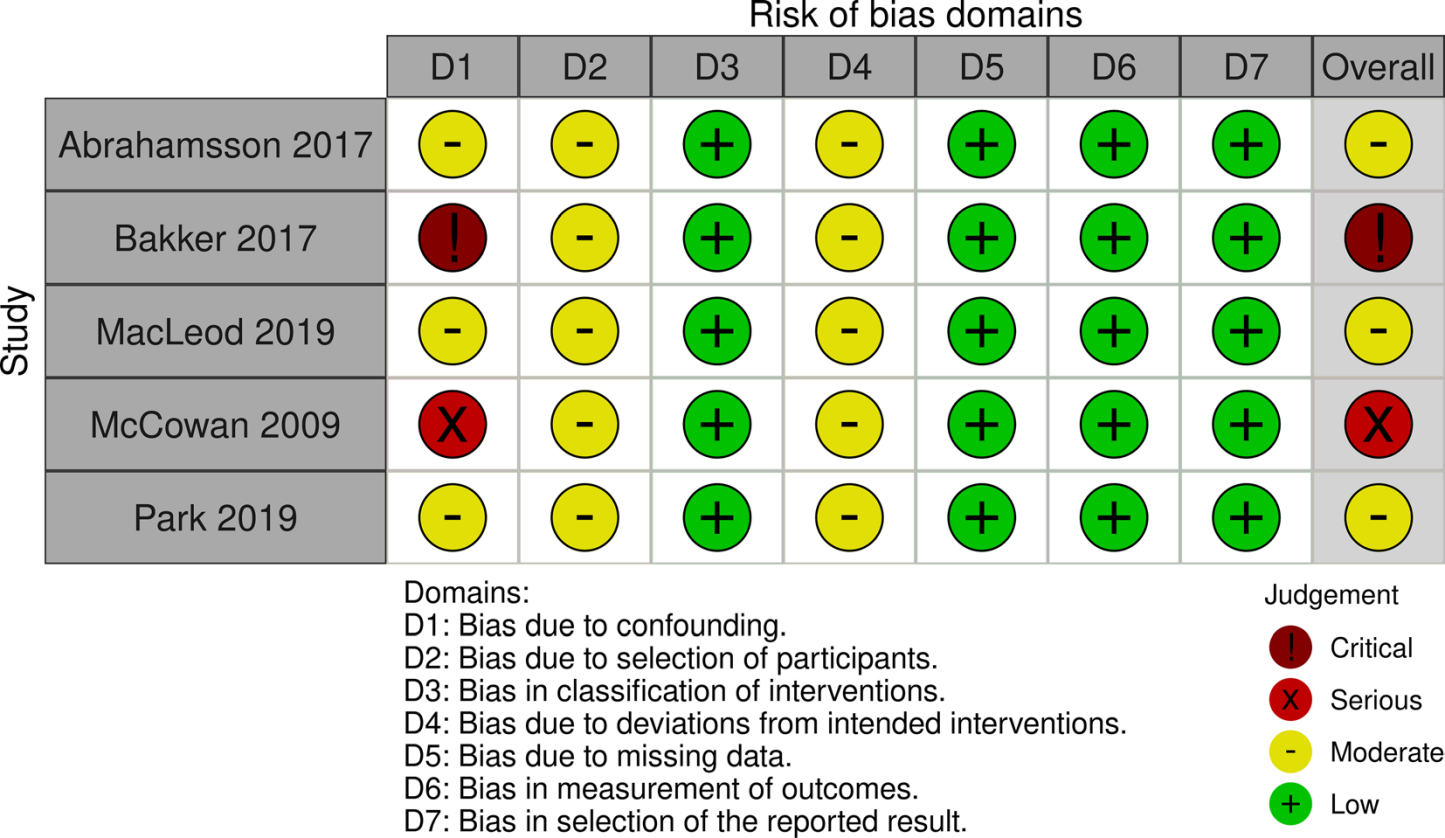
Risk of bias in the included cohort studies

The risk of bias was similar across the different mortality outcomes in all the individual cohort studies. Therefore, the results of the risk of bias assessments are shown at the study level. Three of the five cohort studies had moderate risk of bias, one had serious risk of bias, and one study had critical risk of bias. The main concerns across the studies were risk of bias due to confounding, in selection of participants, and due to uncertainty in whether there were deviations from intended interventions (Fig. 2).

There was moderate risk of bias in the case-control study by Leece and colleagues [41]. Concerns were related to bias due to confounding.

### All-cause mortality

#### All-cause mortality on OAT

**Fig. 3:**
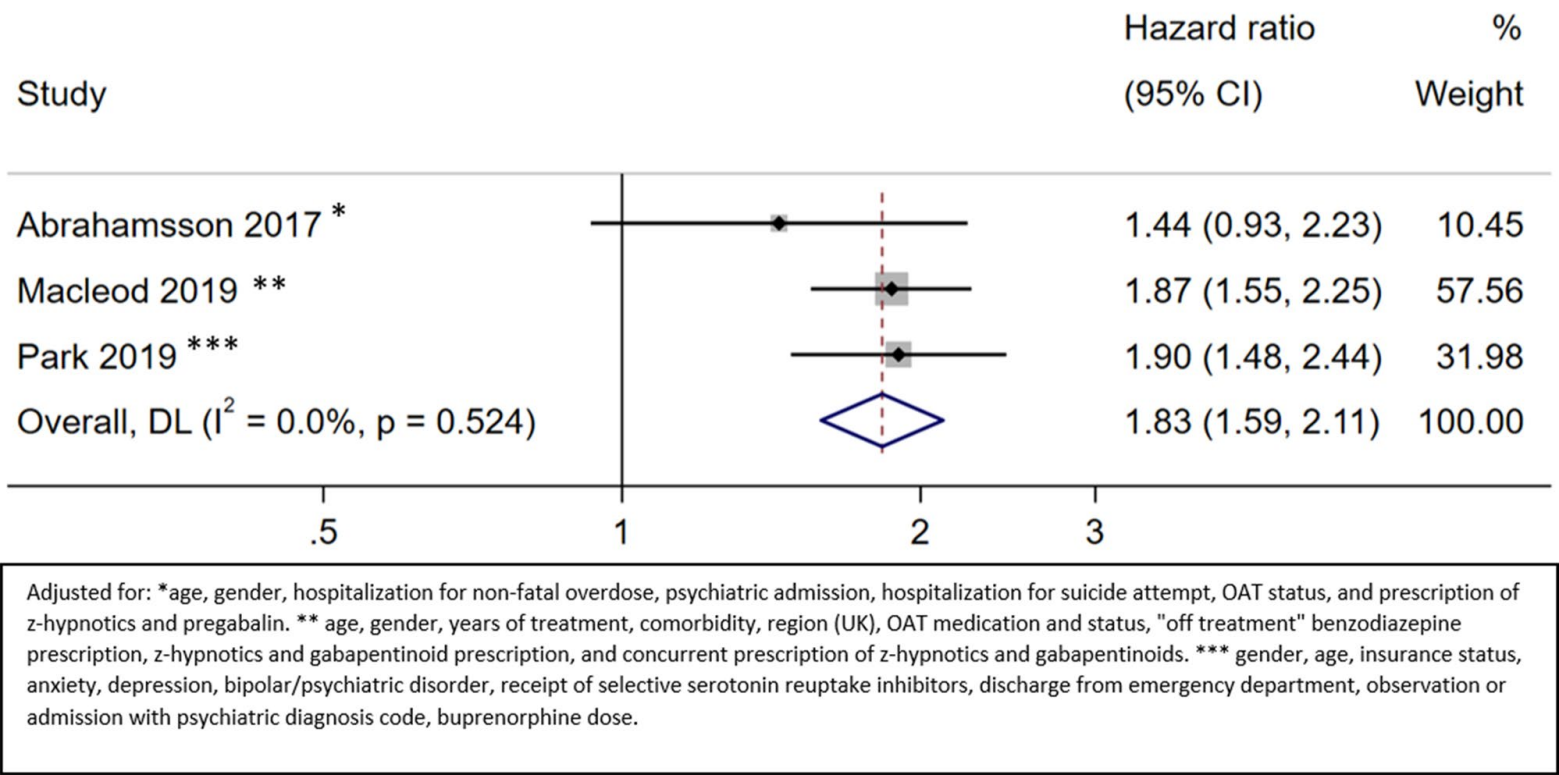
All-cause mortality on OAT

Three studies provided information about all-cause mortality during the OAT period [17, 37, 43]. The HR of all-cause mortality on OAT for patients with benzodiazepine prescription versus no benzodiazepine prescription was 1.83 (95% CI 1.59 to 2.11; moderate certainty) (Fig. 3; Appendix 4). In Macleod 2019 [43], the observation period included up to one year after the end of OAT.

Bakker 2017 [14] also examined the relationship between benzodiazepine prescription and all-cause mortality during OAT but did not report hazard ratios and could therefore not be included in the meta-analysis. The patients were divided into three groups: no benzodiazepine prescription, occasionally prescribed benzodiazepines, and benzodiazepine maintenance treatment. Mortality per one 100 person-years in OAT treatment was 1.31 in the benzodiazepine maintenance treatment group and 1.79 in the group not prescribed benzodiazepines (RR 0.73; 95% CI 0.21 to 3.18; very low certainty) (Appendix 4). We did not estimate RR for the group with occasional benzodiazepine prescription due to the low number of events.

#### All-cause mortality during the whole observation period (on and off OAT)

**Fig. 4:**
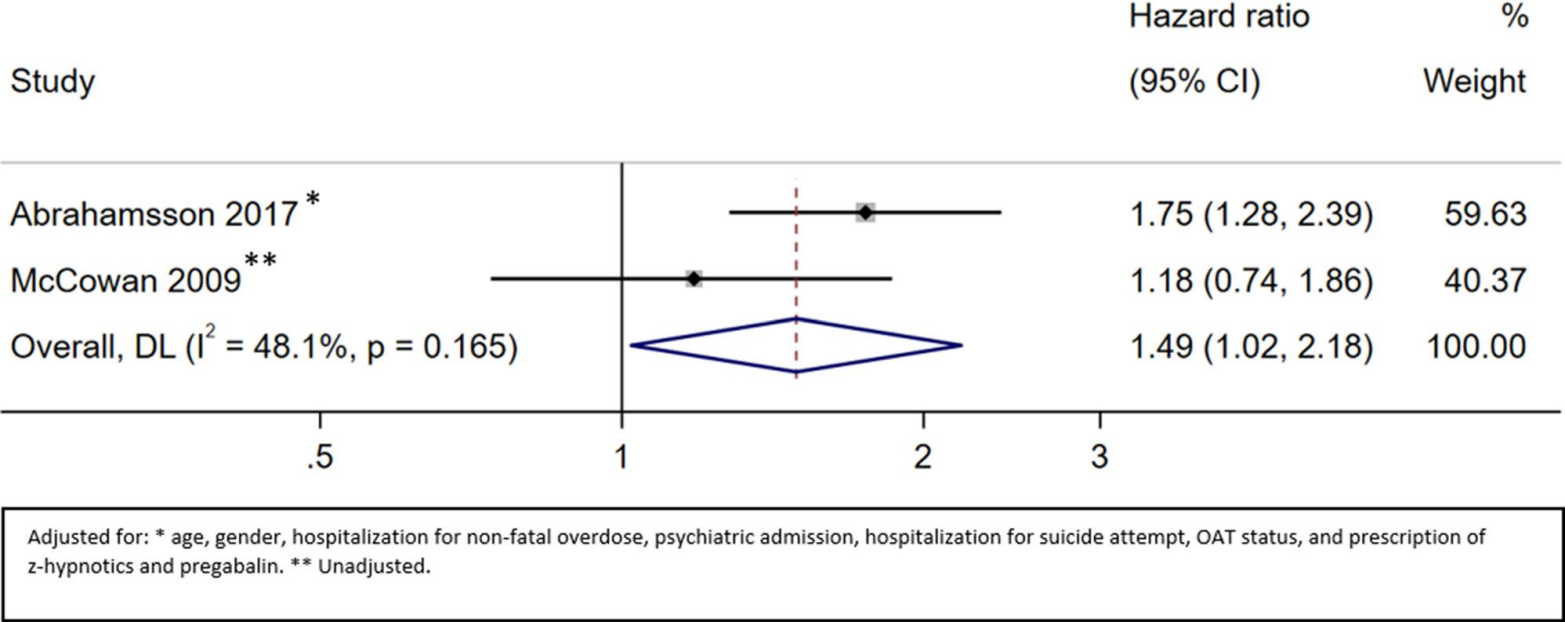
All-cause mortality on and off OAT

Two studies provided information about all-cause mortality throughout the whole observation period [36, 37]. The HR of all-cause mortality during the whole observation period for patients with benzodiazepine prescription versus no benzodiazepine prescription was 1.49 (95% CI 1.02 to 2.18; very low certainty) (Fig. 4, Appendix 4).

### Drug-induced mortality

#### Drug-induced mortality on OAT

Five studies provided information about drug-induced mortality during OAT [14, 17, 37, 41, 43] of which three provided effect estimates that could be pooled in a metanalysis [17, 37, 43]. The results from the two other studies are described separately.

**Fig. 5:**
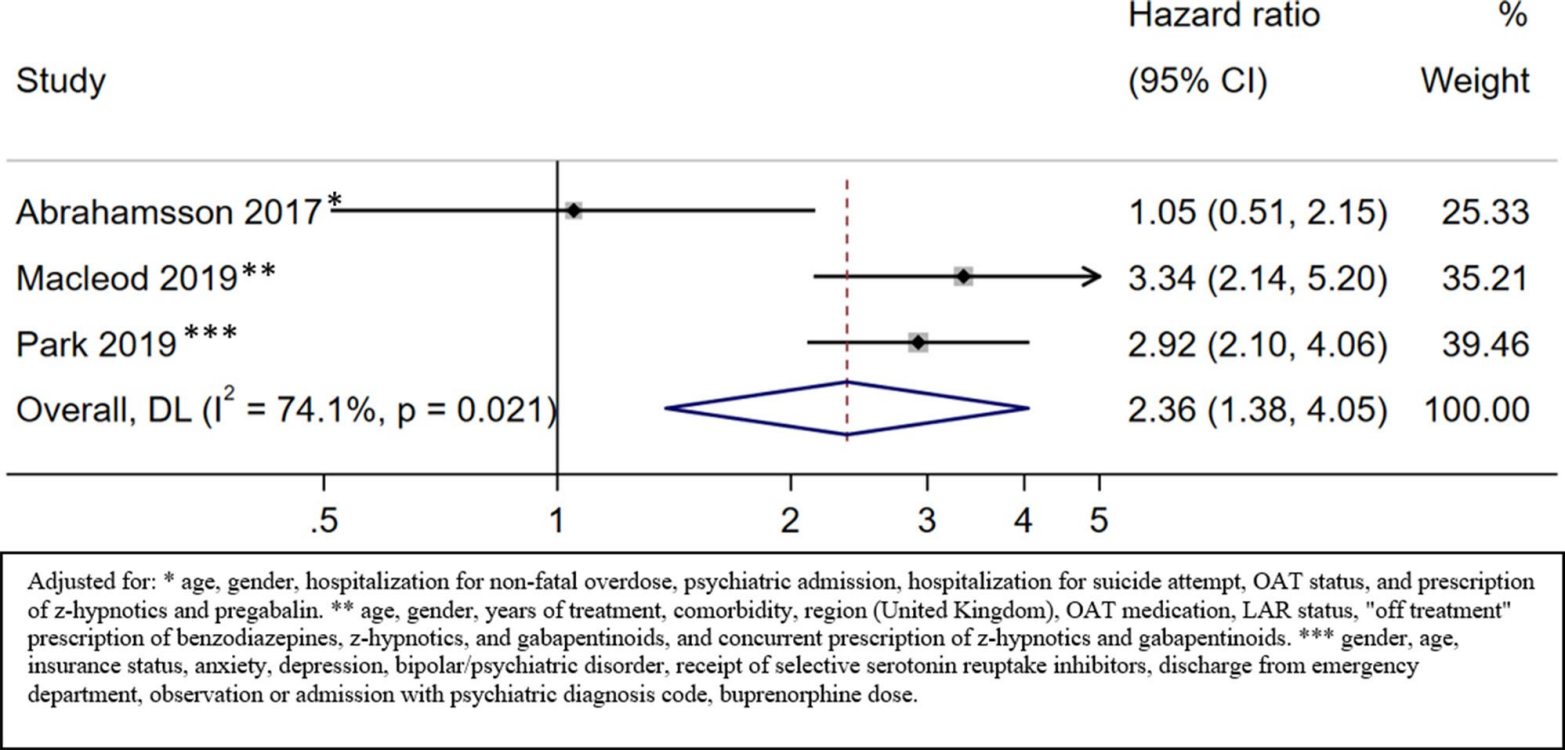
Drug-induced mortality on OAT

The HR of drug-induced mortality on OAT for patients with benzodiazepine prescription versus no benzodiazepine prescription was 2.36 (95% CI 1.38 to 4.05; low certainty) (Fig. 5, Appendix 4).

The case-control study by Leece and colleagues [41] found that 116 of 175 (66.3%) among those who died and 359 of 873 (41.1%) among those who did not die were prescribed benzodiazepines (OR 1.6; 95% CI 1.1 to 2.5; very low certainty) (Appendix 4). The variable for prescription of benzodiazepines was based on prescriptions in the last year before death and it was not specified whether the prescription covered the period when the patient died.

In Bakker 2017 [14], mortality per 100 person-years in treatment was 0.39 in the group that was prescribed benzodiazepines and 0.90 in the group not prescribed benzodiazepines (RR 0.44;95% CI 0.05 to 5.24; very low certainty) (Appendix 4).

#### Drug-induced mortality during the whole observation period (on and off OAT)

**Fig. 6:**
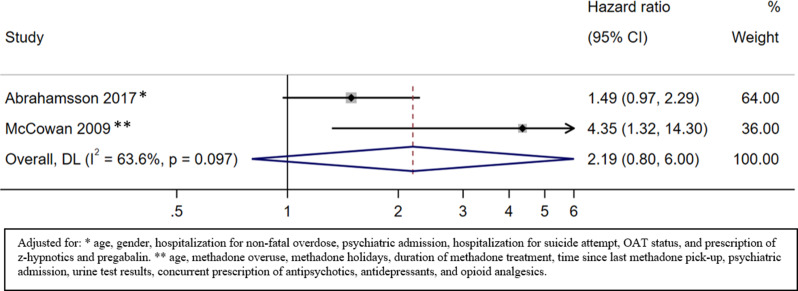
Drug-induced mortality on and off OAT

Two studies presented information about drug-induced mortality throughout the whole observation period [36, 37]. The HR of drug-induced mortality during the whole observation period for patients with benzodiazepine prescription versus no benzodiazepine prescription was 2.19 (95% CI 0.80 to 6.00; very low certainty) (Fig. 6, Appendix 4).

### Mortality due to non-drug-induced causes

#### Mortality due to non-drug induced causes on OAT

**Fig. 7:**
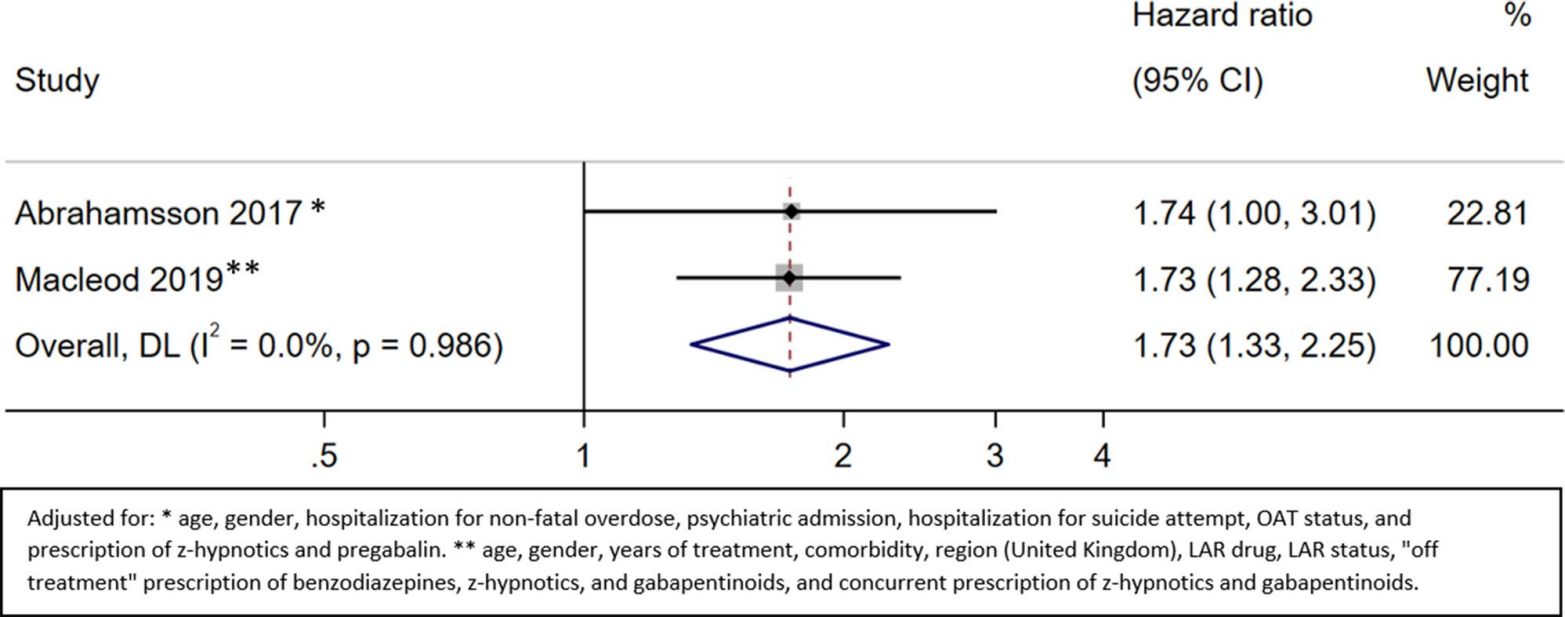
Mortality due to non-drug-induced causes on OAT

Two studies provided information about mortality due to non-drug-induced causes [37, 43]. The HR of mortality due to non-drug-induced causes during OAT for patients with benzodiazepine prescription versus no benzodiazepine prescription was 1.73 (95% CI 1.33 to 2.25; moderate certainty) (Fig. 7, Appendix 4).

#### Mortality due to non-drug-induced causes during the whole observation period (on and off OAT)

**Fig. 8:**
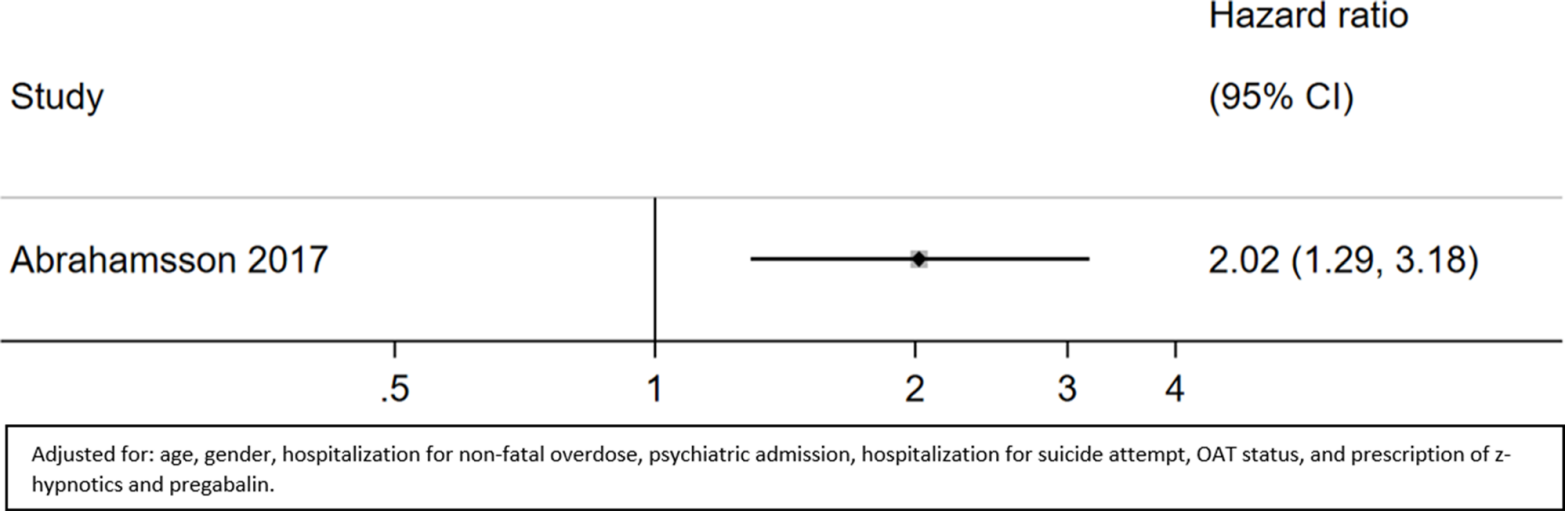
Mortality due to non-drug-induced-causes on and off OAT

One study reported deaths due to non-drug-induced causes throughout the whole observation period [37]. The HR of mortality due to non-drug-induced causes during the whole observation period for patients with benzodiazepine prescription versus no benzodiazepine prescription was 2.02 (95% CI 1.29 to 3.18; moderate certainty) (Fig. 8, Appendix 4).

## Discussion

### Main findings

This review update did not identify any additional evidence beyond what was included in the Norwegian systematic review from 2021 [27]. We identified and included the same six retrospective observational studies (*N* = 84,452) investigating the association between prescription of benzodiazepines to OAT patients and mortality. The studies were conducted in Sweden, Scotland, Canada, England, and the USA, with large variations in structure and content in the OAT programs. Only one of the included studies addressed the prescription of benzodiazepines as maintenance treatment of benzodiazepine dependence, while benzodiazepine dependence status was not specified in the other studies. Five studies were registry-based, and one was a retrospective chart review. The risk of bias was similar across different mortality outcomes in all the included studies. All studies had some methodological limitations and half of them were considered to have a high risk of bias.

Study data did not allow for subgroup analyses of patient, prescriber, or program characteristics, nor of postmortem toxicological data quality.

Moderate certainty evidence suggested prescription was associated with higher all-cause mortality on OAT. Moderate-certainty evidence suggested that benzodiazepine prescription was associated with higher mortality due to non-drug-induced causes both during OAT and throughout the whole observation period (on and off OAT). Low-certainty evidence suggested an association between benzodiazepine prescription and higher drug-induced mortality on OAT. Very low-certainty evidence suggested an association between benzodiazepine prescription and higher all-cause and drug-induced mortality, throughout the entire observation period (on and off OAT).

#### Clinical implications

The study of a possible association between mortality and benzodiazepine co-prescribing to OAT patients is complex and, as demonstrated in this review, the evidence base is weak. Also, leaning on the Scottish experiences [19, 20], efforts to reduce the impact of benzodiazepine prescription on the overdose epidemic, may have adverse and paradoxical consequences. Therefore, it is important to increase the awareness both among OAT patients and prescribers of the potential risks of benzodiazepine use and prescription. Prescribers should be thorough and careful when weighing the pros and cons of benzodiazepine prescribing to OAT patients as described in several recent guidelines [1, 4]. They should assess the patient´s motivations for benzodiazepine use and its possible clinical usefulness and consequences if they decide to prescribe. Further, they should evaluate the effect of prescribing, regarding outcomes such as reduced extra-medical benzodiazepine use and increased stability and quality of life. And finally, benzodiazepine use should not be regarded as a contraindication for OAT [1, 4].

### Implications for research

There is a great need for further, methodologically sound research in this field. Even though the findings of this review suggest an association between benzodiazepine prescription and an increased risk of mortality, the evidence is uncertain. It would therefore be ethically justifiable to conduct randomized trials comparing benzodiazepine prescription with no benzodiazepine prescription in specific OAT patient groups and treatment settings, for instance OAT patients with benzodiazepine co-dependence in specialized treatment clinics. It would be difficult to study mortality in randomized trials, as death is a rare event. In a harm-reduction perspective, however, other outcomes, such as non-prescribed benzodiazepine use and retention on OAT are both valuable and possible to evaluate. Such a study is currently ongoing in Norway [44]. There is also a need for well-designed observational studies of mortality in specific OAT patient groups (e.g., OAT patients with benzodiazepine co-dependence, insomnia and/or anxiety) and treatment settings (e.g., community health care settings versus specialized drug dependence treatment units). For future observational studies, it is particularly important that causes of death are thoroughly reported as well as periods on and off OAT. As drug-induced mortality among opioid dependent people using extra-medical benzodiazepines is a very complex phenomenon and a severe public health problem, it is necessary to develop interventions that will work in real-life contexts. Therefore, research projects addressing this complexity and aiming to develop treatment programs targeting it, including benzodiazepine prescription with harm-reducing intent, are welcome and important [45, 46].

#### Strengths and limitations of this review

The identification of eligible studies for this systematic review is based on an explicit and systematic search strategy. We also consulted topic experts about potentially relevant studies. We may still have missed studies where OAT medications, benzodiazepines and mortality are not mentioned in the title or abstract, but we consider it probable that we have identified all relevant studies. Further, we have assessed the risk of bias and certainty of the evidence transparently and systematically. These assessments allow readers to make their own judgements of the strength of the evidence. Unfortunately, we were not able to express the results from the meta-analyses in absolute measures due to lack of necessary data on exposure group-level in the individual studies.

Finally, to assess the risk of bias from confounding, we identified five key factors—age, sex, comorbidity, psychiatric diagnosis or treatment, and substance use disorder severity—in collaboration with a clinical expert. Nonetheless, we recognize that other confounding factors may also be of importance. Furthermore, because of the observational design, there is a chance for residual confounding due to unmeasured or unknown confounders.

## Conclusions

There is probably an association between prescribed benzodiazepine use and higher risk of all-cause mortality (on OAT) and mortality due to non-drug-induced causes (on OAT and on and off OAT). Benzodiazepines may also be associated with higher all-cause mortality (on and off OAT) and drug-induced mortality (on OAT and on and off OAT), but this is highly uncertain due to methodological issues and possible confounding.

There is a need for future research including well-designed randomized trials and observational studies in different OAT patient groups and treatment settings.

## Electronic supplementary material

Below is the link to the electronic supplementary material.

Supplementary Material 1

## Data Availability

No datasets were generated or analysed during the current study.

## Abbreviations

OAT: Opioid agonist treatment
EUDA: European Union Drugs Agency
GRADE: The Grading of Recommendations Assessment, Development and Evaluation
HR: Hazard Ratio
RR: Risk ratio
CI: Confidence interval
NIPH: Norwegian Institute of Public Health
ROBINS-I: Risk Of Bias In Non-randomized Studies of Interventions
NOS: Newcastle-Ottawa Scale
COSMOS-E: Conducting Systematic Reviews and Meta-Analyses of Observational Studies of Etiology
