# Supplementary material for: The association between benzodiazepine co-prescription, opioid agonist treatment and mortality: a systematic review
Source: BMC Psychiatry. 2024 Oct 28;24:741. doi: 10.1186/s12888-024-06191-3 (PMC11520467; doi:10.1186/s12888-024-06191-3)
Supplement: Supplementary file 1 — Supplementary Material 1 [file 12888_2024_6191_MOESM1_ESM.docx]

# APPENDIXES

# Appendix 1: Search strategy

**Database: Ovid MEDLINE(R) and Epub Ahead of Print, In-Process, In-Data-Review & Other Non-Indexed Citations, Daily and Versions(R) <1946 to June 25, 2021>** 
**Search date: 2021-06-28**

1     "Opiate substitution treatment"/ or "Opioid-Related Disorders"/dt (6045)

2     ((replacement or substitution or agonist or maintenance or medicationassisted or "medication assisted") adj (therap* or treatment* or program*)).ti,ab,kf. (90873)

3     or/1-2 (94236)

4     "Methadone"/ or "Buprenorphine"/ or Morphine/ or methadyl acetate/ or Buprenorphine, Naloxone Drug Combination/ or levomethadone/ (54394)

5     (methadon* or metadon* or Buprenorphin* or Buprenorfin* or levomethadon* or levamethadon* or levametadon* or levometadon* or levadon* or levothyl or "slow release oral morphine" or "sustained relaease oral morphine" or srom).ti,ab,kw,kf. (20169)

6     or/4-5 (60521)

7     exp Benzodiazepines/ (66543)

8     (benzodiazepin* or clonazepam* or diazepam* or Clotiazepam* or Cloxazolam* or Tofizopam* or Bentazepam* or lorazepam* or flurazepam* or nitrazepam* or flunitrazepam* or estazolam* or triazolam* or lormetazepam* or temazepam* or midazolam* or brotizolam* or quazepam* or loprazolam* or doxefazepam* or cinolazepam* or remimazolam* or nimetazepam*).ti,ab,kf. (68799)

9     or/7-8 (95285)

10     Death/ or Mortality/ or exp Drug Overdose/ or Fatal Outcome/ or Mortality, Premature/ (143036)

11     ((benzodiazepin* or clonazepam* or diazepam* or Clotiazepam* or Cloxazolam* or Tofizopam* or Bentazepam* or lorazepam* or flurazepam* or nitrazepam* or flunitrazepam* or estazolam* or triazolam* or lormetazepam* or temazepam* or midazolam* or brotizolam* or quazepam* or loprazolam* or doxefazepam* or cinolazepam* or remimazolam* or nimetazepam*) adj5 (death* or mortal* or overdos* or over-dos*)).ti,ab,kf. (646)

12     (7 and 10) or 11 (1253)

13     exp Prescriptions/ or (prescrip* or prescrib* or illegal or illicit or concomitant or concurrent or combin* or co-ingest*or coingest*).ti,ab,kf. (2451356)

14     12 and 13 (458)

15     (3 or 6) and 9 (3289)

16     14 or 15 (3677)

**Database: Embase <1974 to 2021 June 25>**  
**Search date: 2021-06-28**

1     *opiate substitution treatment/ or *opiate addiction/dt (4948)

2     ((replacement or substitution or agonist or maintenance or medicationassisted or "medication assisted") adj (therap* or treatment* or program*)).ti,ab,kw. (143647)

3     or/1-2 (146426)

4     *methadone treatment/ or *methadone/ or *methadone plus naloxone/ or *buprenorphine plus naloxone/ or *buprenorphine/ or *morphine/ or *levomethadone/ (61944)

5     (methadon* or metadon* or Buprenorphin* or Buprenorfin* or levomethadon* or levamethadon* or levametadon* or levometadon* or levadon* or levothyl or "slow release oral morphine" or "sustained relaease oral morphine" or srom).ti,ab,kw. (27393)

6     or/4-5 (72764)

7     exp *benzodiazepine/ (8076)

8     (benzodiazepin* or clonazepam* or diazepam* or Clotiazepam* or Cloxazolam* or Tofizopam* or Bentazepam* or lorazepam* or flurazepam* or nitrazepam* or flunitrazepam* or estazolam* or triazolam* or lormetazepam* or temazepam* or midazolam* or brotizolam* or quazepam* or loprazolam* or doxefazepam* or cinolazepam* or remimazolam* or nimetazepam*).ti,ab,kw. (98355)

9     or/7-8 (100538)

10     *death/ or *mortality/ or *drug overdose/ (126093)

11     ((benzodiazepin* or clonazepam* or diazepam* or Clotiazepam* or Cloxazolam* or Tofizopam* or Bentazepam* or lorazepam* or flurazepam* or nitrazepam* or flunitrazepam* or estazolam* or triazolam* or lormetazepam* or temazepam* or midazolam* or brotizolam* or quazepam* or loprazolam* or doxefazepam* or cinolazepam* or remimazolam* or nimetazepam*) adj5 (death* or mortal* or overdos* or over-dos*)).ti,ab,kw. (990)

12     (7 and 10) or 11 (1096)

13     *prescription/ or (prescrip* or prescrib* or illegal or illicit or concomitant or concurrent or combin* or co-ingest* or coingest*).ti,ab,kw. (3233465)

14     12 and 13 (465)

15     (3 or 6) and 9 (3930)

16     14 or 15 (4327)

17     limit 16 to embase (2980)

**Database: APA PsycInfo <1806 to June Week 3 2021>** 
**Search date: 2021-06-28**

1     methadone maintenance/ (3688)

2     ((replacement or substitution or agonist or maintenance or medicationassisted or "medication assisted") adj (therap* or treatment* or program*)).tw,id. (12624)

3     or/1-2 (14208)

4     methadone/ or buprenorphine/ or morphine/ (10378)

5     (methadon* or metadon* or Buprenorphin* or Buprenorfin* or levomethadon* or levamethadon* or levametadon* or levometadon* or levadon* or levothyl or "slow release oral morphine" or "sustained relaease oral morphine" or srom).tw,id. (9776)

6     or/4-5 (16458)

7     exp benzodiazepines/ (10936)

8     (benzodiazepin* or clonazepam* or diazepam* or Clotiazepam* or Cloxazolam* or Tofizopam* or Bentazepam* or lorazepam* or flurazepam* or nitrazepam* or flunitrazepam* or estazolam* or triazolam* or lormetazepam* or temazepam* or midazolam* or brotizolam* or quazepam* or loprazolam* or doxefazepam* or cinolazepam* or remimazolam* or nimetazepam*).tw,id. (18653)

9     or/7-8 (20000)

10     "death and dying"/ or mortality rate/ or mortality risk/ or drug overdoses/ (40367)

11     ((benzodiazepin* or clonazepam* or diazepam* or Clotiazepam* or Cloxazolam* or Tofizopam* or Bentazepam* or lorazepam* or flurazepam* or nitrazepam* or flunitrazepam* or estazolam* or triazolam* or lormetazepam* or temazepam* or midazolam* or brotizolam* or quazepam* or loprazolam* or doxefazepam* or cinolazepam* or remimazolam* or nimetazepam*) adj5 (death* or mortal* or overdos* or over-dos*)).tw,id. (192)

12     (7 and 10) or 11 (246)

13     "prescribing (drugs)"/ or exp Prescriptions/ or (prescrip* or prescrib* or illegal or illicit or concomitant or concurrent or combin* or co-ingest* or coingest*).tw,id. (347609)

14     12 and 13 (146)

15     (3 or 6) and 9 (912)

16     14 or 15 (1034)

**Database: The Cochrane Library** 
**Search date: 2021-06-28**

#1 MeSH descriptor: [Opiate Substitution Treatment] explode all trees 328

#2 MeSH descriptor: [Opioid-Related Disorders] explode all trees and with qualifier(s): [drug therapy - DT] 623

#3 ((replacement or substitution or agonist or maintenance or medicationassisted or "medication assisted") NEXT (therap* or treatment* or program*)):ti,ab,kw 24276

#4 #1 or #2 or #3 24638

#5 MeSH descriptor: [Methadone] explode all trees 1284

#6 MeSH descriptor: [Buprenorphine] explode all trees 1176

#7 MeSH descriptor: [Buprenorphine, Naloxone Drug Combination] explode all trees 151

#8 MeSH descriptor: [Morphine] explode all trees 5076

#9 (methadon* or metadon* or Buprenorphin* or Buprenorfin* or levomethadon* or levamethadon* or levametadon* or levometadon* or levadon* or levothyl or "slow release oral morphine" or "sustained relaease oral morphine" or srom):ti,ab,kw 5083

#10 #5 or #6 or #7 or #8 or #9 9909

#11 MeSH descriptor: [Benzodiazepines] explode all trees 9739

#12 (benzodiazepin* or clonazepam* or diazepam* or Clotiazepam* or Cloxazolam* or Tofizopam* or Bentazepam* or lorazepam* or flurazepam* or nitrazepam* or flunitrazepam* or estazolam* or triazolam* or lormetazepam* or temazepam* or midazolam* or brotizolam* or quazepam* or loprazolam* or doxefazepam* or cinolazepam* or remimazolam* or nimetazepam*):ti,ab,kw 20239

#13 #11 or #12 21558

#14 (#4 or #10) and #13 719

#15 MeSH descriptor: [Death] this term only 201

#16 MeSH descriptor: [Mortality] explode all trees 13514

#17 MeSH descriptor: [Drug Overdose] explode all trees 169

#18 #15 or #16 or #17 13867

#19 ((benzodiazepin* or clonazepam* or diazepam* or Clotiazepam* or Cloxazolam* or Tofizopam* or Bentazepam* or lorazepam* or flurazepam* or nitrazepam* or flunitrazepam* or estazolam* or triazolam* or lormetazepam* or temazepam* or midazolam* or brotizolam* or quazepam* or loprazolam* or doxefazepam* or cinolazepam* or remimazolam* or nimetazepam*) NEAR/5 (death* or mortal* or overdos* or over-dos*)):ti,ab,kw 44

#20 (#11 and #18) or #19 68

#21 MeSH descriptor: [Prescriptions] explode all trees 1040

#22 (prescrip* or prescrib* or illegal or illicit or concomitant or concurrent or combin* or co-ingest* or coingest*):ti,ab,kw 321956

#23 #21 or #22 322194

#24 #20 and #23 22

#25 #14 or #24 in Cochrane Reviews 13

#26 ((replacement or substitution or agonist or maintenance or medicationassisted or "medication assisted") NEXT (therap* or treatment* or program*)) 25545

#27 (methadon* or metadon* or Buprenorphin* or Buprenorfin* or levomethadon* or levamethadon* or levametadon* or levometadon* or levadon* or levothyl or "slow release oral morphine" or "sustained relaease oral morphine" or srom) 5214

#28 (benzodiazepin* or clonazepam* or diazepam* or Clotiazepam* or Cloxazolam* or Tofizopam* or Bentazepam* or lorazepam* or flurazepam* or nitrazepam* or flunitrazepam* or estazolam* or triazolam* or lormetazepam* or temazepam* or midazolam* or brotizolam* or quazepam* or loprazolam* or doxefazepam* or cinolazepam* or remimazolam* or nimetazepam*) 20737

#29 ((benzodiazepin* or clonazepam* or diazepam* or Clotiazepam* or Cloxazolam* or Tofizopam* or Bentazepam* or lorazepam* or flurazepam* or nitrazepam* or flunitrazepam* or estazolam* or triazolam* or lormetazepam* or temazepam* or midazolam* or brotizolam* or quazepam* or loprazolam* or doxefazepam* or cinolazepam* or remimazolam* or nimetazepam*) NEAR/5 (death* or mortal* or overdos* or over-dos*)) 78

#30 (prescrip* or prescrib* or illegal or illicit or concomitant or concurrent or combin* or co-ingest* or coingest*) 331392

#31 #1 or #2 or #26 25905

#32 #5 or #6 or #7 or #8 or #27 10031

#33 #11 or #28 22053

#34 (#31 or #32) and #33 851

#35 (#11 and #18) or #29 92

#36 #21 or #30 331624

#37 #35 and #36 45

#38 #34 or #37 in Cochrane Protocols, Trials 744

#39 #25 or #38 757

**Database: Epistemonikos** 
**Search date: 2021-06-28**

((((replacement OR substitution OR agonist OR maintenance OR medicationassisted OR "medication assisted") AND (therap* OR treatment* OR program*)) OR (methadon* OR metadon* OR Buprenorphin* OR Buprenorfin* OR levomethadon* OR levamethadon* OR levametadon* OR levometadon* OR levadon* OR levothyl OR "slow release oral morphine" OR "sustained relaease oral morphine" OR srom)) AND (benzodiazepin* OR clonazepam* OR diazepam* OR Clotiazepam* OR Cloxazolam* OR Tofizopam* OR Bentazepam* OR lorazepam* OR flurazepam* OR nitrazepam* OR flunitrazepam* OR estazolam* OR triazolam* OR lormetazepam* OR temazepam* OR midazolam* OR brotizolam* OR quazepam* OR loprazolam* OR doxefazepam* OR cinolazepam* OR remimazolam* OR nimetazepam*)) : 6 broad synthesis, 70 systematic reviews

((benzodiazepin* OR clonazepam* OR diazepam* OR Clotiazepam* OR Cloxazolam* OR Tofizopam* OR Bentazepam* OR lorazepam* OR flurazepam* OR nitrazepam* OR flunitrazepam* OR estazolam* OR triazolam* OR lormetazepam* OR temazepam* OR midazolam* OR brotizolam* OR quazepam* OR loprazolam* OR doxefazepam* OR cinolazepam* OR remimazolam* OR nimetazepam*) AND (death* OR mortal* OR overdos* OR "over-dose" OR "over-doses" OR "over-dosed" OR "over-dosing")) : 3 broad synthesis, 1 structured summary, 151 systematic reviews

**Description of OpenAlex search**

Search 20.01.2023: OpenAlex via Eppi Reviewer: The six controlled studies from the previous report were used as a basis to search from 01.01. 2021- 20.01. 2023. The search returned 132 references.

**Database: Ovid MEDLINE(R) and Epub Ahead of Print, In-Process, In-Data-Review & Other Non-Indexed Citations, Daily and Versions <1946 to February 05, 2024>**
**Search Strategy:**
1 "Opiate substitution treatment"/ or "Opioid-Related Disorders"/dt (9741)
2 ((replacement or substitution or agonist or maintenance or medicationassisted or "medication assisted") adj (therap* or treatment* or program*)).ti,ab,kf. (105792)
3 or/1-2 (112063)
4 "Methadone"/ or "Buprenorphine"/ or Morphine/ or methadyl acetate/ or Buprenorphine, Naloxone Drug Combination/ or levomethadone/ (58626)
5 (methadon* or metadon* or Buprenorphin* or Buprenorfin* or levomethadon* or levamethadon* or levametadon* or levometadon* or levadon* or levothyl or "slow release oral morphine" or "sustained relaease oral morphine" or srom).ti,ab,kw,kf. (23178)
6 or/4-5 (65477)
7 exp Benzodiazepines/ (70277)
8 (benzodiazepin* or clonazepam* or diazepam* or Clotiazepam* or Cloxazolam* or Tofizopam* or Bentazepam* or lorazepam* or flurazepam* or nitrazepam* or flunitrazepam* or estazolam* or triazolam* or lormetazepam* or temazepam* or midazolam* or brotizolam* or quazepam* or loprazolam* or doxefazepam* or cinolazepam* or remimazolam* or nimetazepam* or oxazepam* or alprazolam*).ti,ab,kf. (76472)
9 or/7-8 (102702)
10 Death/ or Mortality/ or exp Drug Overdose/ or Fatal Outcome/ or Mortality, Premature/ (152360)
11 ((benzodiazepin* or clonazepam* or diazepam* or Clotiazepam* or Cloxazolam* or Tofizopam* or Bentazepam* or lorazepam* or flurazepam* or nitrazepam* or flunitrazepam* or estazolam* or triazolam* or lormetazepam* or temazepam* or midazolam* or brotizolam* or quazepam* or loprazolam* or doxefazepam* or cinolazepam* or remimazolam* or nimetazepam* or oxazepam* or alprazolam*) adj5 (death* or mortal* or overdos* or over-dos*)).ti,ab,kf. (797)
12 (7 and 10) or 11 (1470)
13 exp Prescriptions/ or (prescrip* or prescrib* or illegal or illicit or concomitant or concurrent or combin* or co-ingest*or coingest*).ti,ab,kf. (2936688)
14 12 and 13 (579)
15 (3 or 6) and 9 (3660)
16 14 or 15 (4149)

**Database: Embase <1974 to 2024 February 05>**
**Search Strategy:**
1 *opiate substitution treatment/ or *opiate addiction/dt (7060)
2 ((replacement or substitution or agonist or maintenance or medicationassisted or "medication assisted") adj (therap* or treatment* or program*)).ti,ab,kw. (166571)
3 or/1-2 (171103)
4 *methadone treatment/ or *methadone/ or *methadone plus naloxone/ or *buprenorphine plus naloxone/ or *buprenorphine/ or *morphine/ or *levomethadone/ (65711)
5 (methadon* or metadon* or Buprenorphin* or Buprenorfin* or levomethadon* or levamethadon* or levametadon* or levometadon* or levadon* or levothyl or "slow release oral morphine" or "sustained relaease oral morphine" or srom).ti,ab,kw. (31750)
6 or/4-5 (78644)
7 exp *benzodiazepine/ (8530)
8 (benzodiazepin* or clonazepam* or diazepam* or Clotiazepam* or Cloxazolam* or Tofizopam* or Bentazepam* or lorazepam* or flurazepam* or nitrazepam* or flunitrazepam* or estazolam* or triazolam* or lormetazepam* or temazepam* or midazolam* or brotizolam* or quazepam* or loprazolam* or doxefazepam* or cinolazepam* or remimazolam* or nimetazepam* or oxazepam* or alprazolam*).ti,ab,kw. (111011)
9 or/7-8 (113218)
10 *death/ or *mortality/ or *drug overdose/ (149458)
11 ((benzodiazepin* or clonazepam* or diazepam* or Clotiazepam* or Cloxazolam* or Tofizopam* or Bentazepam* or lorazepam* or flurazepam* or nitrazepam* or flunitrazepam* or estazolam* or triazolam* or lormetazepam* or temazepam* or midazolam* or brotizolam* or quazepam* or loprazolam* or doxefazepam* or cinolazepam* or remimazolam* or nimetazepam* or oxazepam* or alprazolam*) adj5 (death* or mortal* or overdos* or over-dos*)).ti,ab,kw. (1284)
12 (7 and 10) or 11 (1392)
13 *prescription/ or (prescrip* or prescrib* or illegal or illicit or concomitant or concurrent or combin* or co-ingest* or coingest*).ti,ab,kw. (3882807)
14 12 and 13 (621)
15 (3 or 6) and 9 (4512)
16 14 or 15 (5037)
17 limit 16 to embase (3355)

**Database: APA PsycInfo <1806 to January Week 5 2024>**
**Search Strategy:**
1 methadone maintenance/ (3908)
2 ((replacement or substitution or agonist or maintenance or medicationassisted or "medication assisted") adj (therap* or treatment* or program*)).tw,id. (13972)
3 or/1-2 (15617)
4 methadone/ or buprenorphine/ or morphine/ (11351)
5 (methadon* or metadon* or Buprenorphin* or Buprenorfin* or levomethadon* or levamethadon* or levametadon* or levometadon* or levadon* or levothyl or "slow release oral morphine" or "sustained relaease oral morphine" or srom).tw,id. (10881)
6 or/4-5 (17850)
7 exp benzodiazepines/ (11491)
8 (benzodiazepin* or clonazepam* or diazepam* or Clotiazepam* or Cloxazolam* or Tofizopam* or Bentazepam* or lorazepam* or flurazepam* or nitrazepam* or flunitrazepam* or estazolam* or triazolam* or lormetazepam* or temazepam* or midazolam* or brotizolam* or quazepam* or loprazolam* or doxefazepam* or cinolazepam* or remimazolam* or nimetazepam* or oxazepam* or alprazolam*).tw,id. (20691)
9 or/7-8 (21528)
10 "death and dying"/ or mortality rate/ or mortality risk/ or drug overdoses/ (47168)
11 ((benzodiazepin* or clonazepam* or diazepam* or Clotiazepam* or Cloxazolam* or Tofizopam* or Bentazepam* or lorazepam* or flurazepam* or nitrazepam* or flunitrazepam* or estazolam* or triazolam* or lormetazepam* or temazepam* or midazolam* or brotizolam* or quazepam* or loprazolam* or doxefazepam* or cinolazepam* or remimazolam* or nimetazepam* or oxazepam* or alprazolam*) adj5 (death* or mortal* or overdos* or over-dos*)).tw,id. (232)
12 (7 and 10) or 11 (303)
13 "prescribing (drugs)"/ or exp Prescriptions/ or (prescrip* or prescrib* or illegal or illicit or concomitant or concurrent or combin* or co-ingest* or coingest*).tw,id. (390856)
14 12 and 13 (180)
15 (3 or 6) and 9 (1000)
16 14 or 15 (1153)

**Database: Cochrane Library**
**Search date: 2024-02-08**

#1 MeSH descriptor: [Opiate Substitution Treatment] explode all trees 518

#2 MeSH descriptor: [Opioid-Related Disorders] explode all trees and with qualifier(s): [drug therapy - DT] 1083

#3 ((replacement or substitution or agonist or maintenance or medicationassisted or "medication assisted") NEXT (therap* or treatment* or program*)):ti,ab,kw 28790

#4 #1 or #2 or #3 29434

#5 MeSH descriptor: [Methadone] explode all trees 1621

#6 MeSH descriptor: [Buprenorphine] explode all trees 1560

#7 MeSH descriptor: [Buprenorphine, Naloxone Drug Combination] explode all trees 224

#8 MeSH descriptor: [Morphine] explode all trees 5995

#9 (methadon* or metadon* or Buprenorphin* or Buprenorfin* or levomethadone or levamethadone or levametadon* or levometadon* or levadone or levothyl or "slow release oral morphine" or "sustained relaease oral morphine" or srom):ti,ab,kw 5836

#10 #5 or #6 or #7 or #8 or #9 11532

#11 MeSH descriptor: [Benzodiazepines] explode all trees 11649

#12 (benzodiazepin* or clonazepam or diazepam or Clotiazepam or Cloxazolam or Tofizopam or Bentazepam or lorazepam or flurazepam or nitrazepam or flunitrazepam or estazolam or triazolam or lormetazepam or temazepam or midazolam or brotizolam or quazepam or loprazolam or doxefazepam or cinolazepam or remimazolam or nimetazepam or oxazepam or alprazolam):ti,ab,kw 23906

#13 #11 or #12 24977

#14 (#4 or #10) and #13 825

#15 MeSH descriptor: [Death] this term only 388

#16 MeSH descriptor: [Mortality] explode all trees 18841

#17 MeSH descriptor: [Drug Overdose] 2 tree(s) exploded 0

#18 #15 or #16 or #17 19212

#19 ((benzodiazepin* or clonazepam or diazepam or Clotiazepam or Cloxazolam or Tofizopam or Bentazepam or lorazepam or flurazepam or nitrazepam or flunitrazepam or estazolam or triazolam or lormetazepam or temazepam or midazolam or brotizolam or quazepam or loprazolam or doxefazepam or cinolazepam or remimazolam or nimetazepam or oxazepam or alprazolam) NEAR/5 (death* or mortal* or overdos* or over-dos*)):ti,ab,kw 48

#20 (#11 and #18) or #19 72

#21 MeSH descriptor: [Prescriptions] explode all trees 1579

#22 (prescrip* or prescrib* or illegal or illicit or concomitant or concurrent or combin* or co-ingest*):ti,ab,kw 390042

#23 #21 or #22 390363

#24 #20 and #23 23

#25 #14 or #24 in Cochrane Reviews 14

#26 ((replacement or substitution or agonist or maintenance or medicationassisted or "medication assisted") NEXT (therap* or treatment* or program*)) 30462

#27 (methadon* or metadon* or Buprenorphin* or Buprenorfin* or levomethadone or levamethadone or levametadon* or levometadon* or levadone or levothyl or "slow release oral morphine" or "sustained relaease oral morphine" or srom) 5981

#28 (benzodiazepin* or clonazepam or diazepam or Clotiazepam or Cloxazolam or Tofizopam or Bentazepam or lorazepam or flurazepam or nitrazepam or flunitrazepam or estazolam or triazolam or lormetazepam or temazepam or midazolam or brotizolam or quazepam or loprazolam or doxefazepam or cinolazepam or remimazolam or nimetazepam or oxazepam or alprazolam) 23519

#29 ((benzodiazepin* or clonazepam or diazepam or Clotiazepam or Cloxazolam or Tofizopam or Bentazepam or lorazepam or flurazepam or nitrazepam or flunitrazepam or estazolam or triazolam or lormetazepam or temazepam or midazolam or brotizolam or quazepam or loprazolam or doxefazepam or cinolazepam or remimazolam or nimetazepam or oxazepam or alprazolam) NEAR/5 (death* or mortal* or overdos* or over-dos*)) 93

#30 (prescrip* or prescrib* or illegal or illicit or concomitant or concurrent or combin* or co-ingest*) 400795

#31 #1 or #2 or #26 31103

#32 #5 or #6 or #7 or #8 or #27 11664

#33 #11 or #28 25060

#34 (#31 or #32) and #33 962

#35 (#11 and #18) or #29 108

#36 #21 or #30 401110

#37 #35 and #36 55

#38 #34 or #37 in Cochrane Protocols, Trials 845

#39 #25 or #38 870

**Database: Epistemonikos**
**Søkedato: 2024-02-08**
**Søk i title/abstract**

((((replacement OR substitution OR agonist OR maintenance OR medicationassisted OR "medication assisted") AND (therap* OR treatment* OR program*)) OR (methadon* OR metadon* OR Buprenorphin* OR Buprenorfin* OR levomethadon* OR levamethadon* OR levametadon* OR levometadon* OR levadon* OR levothyl OR "slow release oral morphine" OR "sustained relaease oral morphine" OR srom)) AND (benzodiazepin* OR clonazepam* OR diazepam* OR Clotiazepam* OR Cloxazolam* OR Tofizopam* OR Bentazepam* OR lorazepam* OR flurazepam* OR nitrazepam* OR flunitrazepam* OR estazolam* OR triazolam* OR lormetazepam* OR temazepam* OR midazolam* OR brotizolam* OR quazepam* OR loprazolam* OR doxefazepam* OR cinolazepam* OR remimazolam* OR nimetazepam* or oxazepam* or alprazolam*)) : 6 broad syntheses, 88 systematic reviews

(((benzodiazepin* OR clonazepam* OR diazepam* OR Clotiazepam* OR Cloxazolam* OR Tofizopam* OR Bentazepam* OR lorazepam* OR flurazepam* OR nitrazepam* OR flunitrazepam* OR estazolam* OR triazolam* OR lormetazepam* OR temazepam* OR midazolam* OR brotizolam* OR quazepam* OR loprazolam* OR doxefazepam* OR cinolazepam* OR remimazolam* OR nimetazepam* OR oxazepam* OR alprazolam*) AND (death* OR mortal* OR overdos* OR "over-dose" OR "over-doses" OR "over-dosed" OR "over-dosing"))) : 11 broad syntheses, 1 structured summary, 206 systematic reviews

# **Appendix 2:** The included studies’ definitions of the outcome **referred to as drug-induced deaths in this review**

| **Study** | **Term** | **Definition** |
| --- | --- | --- |
| Abrahamsson 2017 | Overdose death | Overdose death included all deaths with codes X40-49 (accidental overdoses) or Y10-19 (overdoses with undetermined intent) registered as the underlying cause of death in the CDR. |
| McCowan 2009 | Drug dependent cause specific mortality | Not defined  Cause of death registered in the GRO death certificate |
| Leece  2015 | Opioid-related deaths | The investigating coroner classified opioid-related deaths as those with toxicologic findings of opioid concentrations sufficiently high to cause death, or that a combination of drugs (including at least one opioid present at a clinically significant concentration) resulted in death |
| Macleod  2019 | Drug related deaths | Se description below* |
| Park  2019 | Opioid overdose | Classification of fatal opioid overdose was based on medical examiner determination or standardized assessment by the MDPH.  The following ICD-10 codes were selected from the underlying cause of death field to identify all poisonings/overdoses: X40-X49, X60-X69, X85-X90, Y10-Y19, and Y35.2. All multiple cause of death fields were then used to identify fatal opioid overdoses: T40.0, T40.1, T40.2, T40.3, T40.4, and T40.6. Additional fatal opioid overdose cases were identified by searching the cause of death text fields for opioid-related terms for deaths occurring in 2014 and 2015 that had not yet received an ICD-10 code. |
| \| **CDR -the Cause of Death Register; MDPH - Massachusetts Department of Public Health; GRO - General Registry Office; ICD-10 - International Classification of Diseases – 10th edition**    ***** Definitions of drug related deaths in Macleod 2019 \| \| \| \| --- \| --- \| --- \| \| **Description** \| **ICD-9 code** \| **ICD-10 code** \| \| Mental and behavioural disorders \|  \|  \| \| due to drug use (excluding alcohol and tobacco) ^a^ \| 292, 304, 305.2–305.9 \| F11–F16, F18–F19 \| \| Unspecified cause/disorder \|  \| F99 \| \| Accidental self-harm \|  \|  \| \| Poisoning by drugs, medicaments and biological substances ^a^ \| E850–E858 \| X40–X44 \| \| Poisoning, other or unspecified exposure \| E866.8, E866.9 \| X49 \| \| Other or unspecified means \| E928.8, E928.9 \| X58, X59.9 \| \| Intentional self-harm \|  \|  \| \| Poisoning by drugs, medicaments and biological substances ^a^ \| E950.0–E950.5 \| X60–X64 \| \| Poisoning, other or unspecified exposure \| E950.9 \| X69 \| \| Other or unspecified means \| E958.8, E958.9 \| X83, X84 \| \| Assault by \|  \|  \| \| Poisoning by drugs, medicaments and biological substances ^a^ \| E962.0 \| X85 \| \| Poisoning, other or unspecified exposure \| E962.9 \| X90 \| \| Other or unspecified means \| E968.8, E968.9 \| Y08, Y09 \| \| Self-harm, undetermined intent \|  \|  \| \| Poisoning by drugs, medicaments and biological substances ^a^ \| E980.0–E980.5 \| Y10–Y14 \| \| Poisoning, other or unspecified exposure \| E980.9 \| Y19 \| \| Other or unspecified means \| E988.8, E988.9 \| Y33, Y34 \| \| External cause \|  \|  \| \| Poisoning by drugs, medicaments and biological substances \| 960-979 \| T36-T50 \| \| Poisoning, other or unspecified exposure \| 989.89, 989.9 \| T65.8, T65.9 \| \| Other or unspecified cause \| 995.89 \| T78.8, T78.9 \| \|  \|  \|  \| \| Ill-defined, unpecified or unknown cause \| 798.1–798.9, 799.89, 799.9 \| R68.8, R69, R96-R99 \| | | |

# Appendix 3: List of excluded studies with reason

**Wrong target group (n = 15)**

Bharat C, Gisev N and Barbieri S ; Dobbins T ; Larney S ; Buizen L ; Degenhardt L ;. 2024. "Prescription opioid use among people with opioid dependence and concurrent benzodiazepine and gabapentinoid exposure: An analysis of overdose and all-cause mortality". *International Journal of Drug Policy* 123:104287.

Bannon Michael J, Lapansie Allyson R; Jaster Alaina M; Saad Manal H; Lenders Jayna and Schmidt Carl J;. (2021). Opioid deaths involving concurrent benzodiazepine use: Assessing risk factors through the analysis of prescription drug monitoring data and postmortem toxicology. *Drug And Alcohol Dependence*, 225, pp.108854-108854.

Calcaterra S L, Severtson S G; Bau G E; Margolin Z R; Bucher-Bartelson B and Green J L; Dart R C;. (2018). Trends in intentional abuse or misuse of benzodiazepines and opioid analgesics and the associated mortality reported to poison centers across the United States from 2000 to 2014. *Clinical Toxicology: The Official Journal of the American Academy of Clinical Toxicology & European Association of Poisons Centres & Clinical Toxicologists*, 56(11), pp.1107-1114.

Cho J, Spence M M and Niu F ; Hui R L; Gray P ; Steinberg S ;. (2020). Risk of Overdose with Exposure to Prescription Opioids, Benzodiazepines, and Non-benzodiazepine Sedative-Hypnotics in Adults: a Retrospective Cohort Study. *Journal of General Internal Medicine*, 35(3), pp.696-703.

Dasgupta N, Funk M J and Proescholdbell S ; Hirsch A ; Ribisl K M; Marshall S ;. (2016). Cohort Study of the Impact of High-Dose Opioid Analgesics on Overdose Mortality. *Pain Medicine*, 17(1), pp.85-98.

Fugelstad Anna, Bremberg Sven and Hjelmström Peter ; Thiblin Ingemar ;. (2021). Methadone‐related deaths among youth and young adults in Sweden 2006–15. *Addiction*, , pp..

Gaither J R, Goulet J L; Becker W C; Crystal S and Edelman E J; Gordon K ; Kerns R D; Rimland D ; Skanderson M ; Justice A C; Fiellin D A;. (2016). The Association Between Receipt of Guideline-Concordant Long-Term Opioid Therapy and All-Cause Mortality. *Journal of General Internal Medicine*, 31(5), pp.492-501.

Hawkins E J and Goldberg S B; Malte C A; Saxon A J;. (2019). New Coprescription of Opioids and Benzodiazepines and Mortality Among Veterans Affairs Patients With Posttraumatic Stress Disorder. *Journal of Clinical Psychiatry*, 80(4), pp.09.

Li K J and Smedberg D L; DeLisi L E;. (2019). A Retrospective 4-year Outcome Study of Veterans Admitted to an Acute Inpatient Detoxification Unit for Opioid Use Disorder. *American Journal on Addictions*, 28(4), pp.318-323.

Mariottini Claudia, Kriikku Pirkko and Ojanperä Ilkka ;. (2021). Concomitant drugs with buprenorphine user deaths. *Drug And Alcohol Dependence*, 218, pp.108345-108345.

Mooney L J, Zhu Y and Yoo C K; Wolitzky-Taylor K ; Hser Y I;. 2022. "Association Between Benzodiazepine and Opioid Prescription and Mortality Among Patients in a Large Healthcare System". *Journal of Addiction Medicine* 16(1):65-71.

Szmulewicz Alejandro G, Bateman Brian T; Levin Raisa and Huybrechts Krista F;. (2021). The Risk of Overdose With Concomitant Use of Z-Drugs and Prescription Opioids: A Population-Based Cohort Study. *American Journal Of Psychiatry*, 178(7), pp.643-650.

Thylstrup B, Seid A K and Tjagvad C ; Hesse M ;. (2020). Incidence and predictors of drug overdoses among a cohort of >10,000 patients treated for substance use disorder. *Drug and Alcohol Dependence*, 206 (no pagination), pp..

Walde J, Andersson L and Johnson B ; Hakansson A ;. 2023. "Drug prescriptions preceding opioid-related deaths-a register study in forensic autopsy patients". *PLoS ONE [Electronic Resource]* 18(5):e0285583.

Yang B R, Oh I S; Li J and Jeon H L; Shin J Y;. (2020). Association between opioid analgesic plus benzodiazepine use and death: A case-crossover study. *Journal of Psychosomatic Research*, 135, pp.110153.

**Wrong intervention (n = 10)**

Bech A B, Clausen T and Waal H ; Vindenes V ; Edvardsen H E; Frost J ; Skeie I ;. (2020). Post-mortem toxicological analyses of blood samples from 107 patients receiving opioid agonist treatment: substances detected and pooled opioid and benzodiazepine concentrations. *Addiction*, 01, pp.01.

Bhatraju Elenore, Fuller Caitlin C and Grekin Paul ; Rockman Shay ; Peavy K Michelle;. (2021). Mortality in an Opioid Treatment Program. *Journal Of Psychoactive Drugs*, , pp..

Caplehorn J R. M. (1996). Risk factors for non-HIV-related death among methadone maintenance patients. *European Addiction Research*, 2(1), pp.49-52.

Chan G M, Stajic M and Marker E K; Hoffman R S; Nelson L S;. (2006). Testing positive for methadone and either a tricyclic antidepressant or a benzodiazepine is associated with an accidental overdose death: analysis of medical examiner data. *Academic Emergency Medicine*, 13(5), pp.543-7.

Jones Nicola R, Hickman Matthew and Nielsen Suzanne ; Larney Sarah ; Dobbins Timothy ; Ali Robert ; Degenhardt Louisa ;. (2022). The impact of opioid agonist treatment on fatal and non-fatal drug overdose among people with a history of opioid dependence in NSW, Australia, 2001–2018: Findings from the OATS retrospective linkage study. *Drug And Alcohol Dependence*, 236, pp.109464-109464.

Peles E, Schreiber S and Adelson M ;. (2010). 15-Year survival and retention of patients in a general hospital-affiliated methadone maintenance treatment (MMT) center in Israel. *Drug and Alcohol Dependence*, 107(2-3), pp.141-148.

Peles E, Kim Y and Sason A ; Adelson M ; Levran O ;. 2023. "Predictors of treatment retention and survival among methadone-maintained patients: A possible role for a functional delta opioid receptor gene variant". *Drug & Alcohol Dependence* 250:110903.

Peles E, Schreiber S and Sason A ; Adelson M ;. (2018). Similarities and changes between 15- and 24-year survival and retention rates of patients in a large medical-affiliated methadone maintenance treatment (MMT) center. *Drug & Alcohol Dependence*, 185, pp.112-119.

Skeie Ivar, Clausen Thomas and Hjemsæter Arne Jan; Landheim Anne Signe; Monsbakken Bent ; Thoresen Magne ; Waal Helge ;. (2022). Mortality, Causes of Death, and Predictors of Death among Patients On and Off Opioid Agonist Treatment: Results from a 19-Year Cohort Study. *European Addiction Research*, 28(5), pp.358-367.

Wolf B C and Lavezzi W A; Sullivan L M; Flannagan L M;. (2004). Methadone-related deaths in Palm Beach County. *Journal of Forensic Sciences*, 49(2), pp.375-8.

**Wrong study design (n = 5)**

Boon M, van Dorp E and Broens S ; Overdyk F ;. (2020). Combining opioids and benzodiazepines: effects on mortality and severe adverse respiratory events. *Annals of Palliative Medicine*, 9(2), pp.542-557.

Brenet O, Harry P and Le Bouil A ; Cailleux A ; Geoffroy S ; Jouan P L; Alquier P ;. (1998). Intoxication-related death due to concomitant treatment with buprenorphine and benzodiazepines. [French]. *Reanimation Urgences*, 7(6), pp.673.

Lintzeris N and Nielsen S . (2010). Benzodiazepines, methadone and buprenorphine: interactions and clinical management. *American Journal on Addictions*, 19(1), pp.59-72.

Reynaud M, Petit G and Potard D ; Courty P ;. (1997). Misuse of buprenorphine-benzodiazepine combinations: Six deaths [1]. [French]. *Presse Medicale*, 26(28), pp.1337-1338.

Yuan W and Williams B N. (2012). Interactions among benzodiazepine and buprenorphine/naloxone. *Journal of Emergency Nursing*, 38(1), pp.5-6.

**Wrong outcome (n = 4)**

Miano Todd A, Wang Lei and Leonard Charles E; Brensinger Colleen M; Acton Emily K; Dawwas Ghadeer K; Bilker Warren B; Soprano Samantha E; Nguyen Thanh Phuong Pham; Woody George ; Yu Elmer ; Neuman Mark ; Li Lang ; Hennessy Sean ;. (2022). Identifying Clinically Relevant Drug–Drug Interactions With Methadone and Buprenorphine: A Translational Approach to Signal Detection. *Clinical Pharmacology & Therapeutics*, 112(5), pp.1120-1129.

Smolina Kate, Crabtree Alexis and Chong Mei ; Park Mina ; Mill Christopher ; Zhao Bin ; Schütz Christian G;. (2022). Prescription-related risk factors for opioid-related overdoses in the era of fentanyl contamination of illicit drug supply: A retrospective case-control study. *Substance Abuse*, 43(1), pp.92-98.

Xu Kevin Y, Borodovsky Jacob T; Presnall Ned and Mintz Carrie M; Hartz Sarah M; Bierut Laura J; Grucza Richard A;. (2021). Association Between Benzodiazepine or Z-Drug Prescriptions and Drug-Related Poisonings Among Patients Receiving Buprenorphine Maintenance: A Case-Crossover Analysis. *American Journal Of Psychiatry*, 178(7), pp.651-659.

Xu K Y, Borodovsky J T; Presnall N and Mintz C M; Hartz S M; Bierut L J; Grucza R A;. (2021). Association Between Benzodiazepine or Z-Drug Prescriptions and Drug-Related Poisonings Among Patients Receiving Buprenorphine Maintenance: A Case-Crossover Analysis. *American Journal of Psychiatry*, , pp.appiajp202020081174.

# Appendix 4: Evidence profiles (Grade assessments)

**Author(s):** Abrahamsson 2017, Bakker 2017, Macleod 2019, McCowan 2009, Leece 2015, Park 2019

**Question:** Prescription of benzodiazepines compared to no prescription of benzodiazepines in opioid maintenance treatment (OAT)

**Setting:** OAT

| **Certainty assessment** | | | | | | | **№ of patients** | | **Effect** | | | **Certainty** | **Importance** |
| --- | --- | --- | --- | --- | --- | --- | --- | --- | --- | --- | --- | --- | --- |
| **№ of studies** | **Study design** | **Risk of bias** | **Inconsistency** | **Indirectness** | **Imprecision** | **Other considerations** | **Prescription of benzodiazepines** | **no prescription of benzodiazepines** | **Relative (95% CI)** | | **Absolute (95% CI)** |  |  |
| **All-cause mortality on OAT** | | | | | | | | | | | | | |
| 3 | observational studies | serious^a^ | not serious | not serious | not serious | none | - | - | **HR 1.83** (1.59 to 2.11) | | **-** | ⨁⨁⨁◯ Moderate |  |
| **All-cause mortality on and off OAT** | | | | | | | | | | | | | |
| 2 | observational studies | very serious^b^ | serious^d^ | not serious | not serious | none | - | - | **HR 1.49** (1.02 to 2.18) | | **-** | ⨁◯◯◯ Very low |  |
| **Drug-induced mortality on OAT** | | | | | | | | | | | | | |
| 3 | observational studies | serious^a^ | Serious^d^ | not serious | not serious | none | - | - | **HR 2.36** (1.38 to 4.05) | | **-** | ⨁⨁◯◯ Low |  |
| **Drug-induced mortality on and off OAT** | | | | | | | | | | | | | |
| 2 | observational studies | very serious^b^ | serious ^d^ | not serious | serious^e^ | none | - | - | **HR 2.19** (0.80 to 6.00) | | **-** | ⨁◯◯◯ Very low |  |
| **Mortality due to other causes on OAT** | | | | | | | | | | | | | |
| 2 | observational studies | serious^a^ | not serious | not serious | not serious | none | - | - | **HR 1.73** (1.33 to 2.25) | | **-** | ⨁⨁⨁◯ Moderate |  |
| **Mortality due to other causes on and off OAT** | | | | | | | | | | | | | |
| 1 | observational study | serious^a^ | not serious | not serious | not serious | none | - | - | **HR 2.02** (1.29 to 3.18) | | **-** | ⨁⨁⨁◯ Moderate |  |
| **Drug-induced mortality on OAT (case-control study)** | | | | | | | | | | | | | |
| 1 | observational study | serious^a^ | not serious | not serious | serious^e^ | none | 175 cases 873 controls | | **OR 1.6** (1.1 to 2.5) | | - | ⨁◯◯◯ Very low |  |
| **All-cause mortality on OAT (Bakker 2017)** | | | | | | | | | |  |  |  |  |
| 1 | observational study | extremely serious ^c^ | not serious | not serious | serious^e^ | none | - | - | **RR 0.73**  (0,21 til 3,18) | | - | ⨁◯◯◯ Very low |  |

**Drug-induced mortality (Bakker 2017)**

| 1 | observational study | extremely serious^c^ | not serious | not serious | Serious^e^ | none | - | - | **RR 0.44**  (0.05-5.24) | - | ⨁◯◯◯ Very low |  |
| --- | --- | --- | --- | --- | --- | --- | --- | --- | --- | --- | --- | --- |

**CI:** confidence interval; **HR:** hazard Ratio; **OR:** odds ratio; RR: risk ratio

#### Explanations

a. Downgraded due to moderate risk of bias due to confounding, moderate risk of selection bias and moderate risk of deviations from intended intervention.

b. Downgraded due to serious risk of bias due to confounding, moderate risk of selection bias and moderate risk of deviations from intended intervention

c. Downgraded due to critical risk of bias due to confounding, moderate risk of selection bias and moderate risk of deviations from intended intervention

d. Downgraded due to inconsistency between studies

e. Downgraded due to wide confidence interval
